# 2,5-Bis[(3-hy­droxy­prop­yl)amino]-1,4-benzoquinone monohydrate

**DOI:** 10.1107/S1600536811021635

**Published:** 2011-06-11

**Authors:** Jian Wang, Weiwei Liu, Tongwei Guan, Fujun Yin, Yuxi Sun

**Affiliations:** a School of Chemical Engineering, Huaihai Institute of Technology, Lianyungang Jiangsu, People’s Repubic of China; b Qufu Normal University, Qufu Shandong, People’s Repubic of China

## Abstract

The title compound, C_12_H_18_N_2_O_4_·H_2_O, was obtained as a product of the reaction of hydro­quinone with *n*-propanol amine. The compound crystallizes as a monohydrate, integrating water into its hydrogen-bonded network. Each diamino­quinone moiety forms two centrosymmetric 10-membered rings through C=O⋯H—N bonds. The resulting bands along [102] are inter­linked through hy­droxy groups and water mol­ecules into three-dimensional network. The chemically equivalent bond lengths in the diamino­quinone moiety exhibit a perceptible discrepancy [*e.g.* C=O bond lengths differ by 0.016 (2) Å], apparently as a result of asymmetric hydrogen bonding: one O atom serves as an acceptor of one hydrogen bond, whereas the other is an acceptor of two.

## Related literature

For the synthesis of the title compound see: Jian *et al.* (2009 ▶). For related literature on aminoquinones, see: Der (2010 ▶), Nisha *et al.* (2010 ▶).
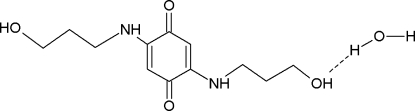


## Experimental

### 

#### Crystal data

C_12_H_18_N_2_O_4_·H_2_O
*M* *_r_* = 272.30
Triclinic, 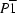

*a* = 4.9272 (8) Å
*b* = 11.673 (2) Å
*c* = 11.933 (2) Å
α = 82.104 (2)°
β = 87.994 (2)°
γ = 80.849 (2)°
*V* = 671.13 (19) Å^3^
*Z* = 2
Mo *K*α radiation
μ = 0.11 mm^−1^
*T* = 296 K
0.25 × 0.18 × 0.11 mm

#### Data collection

Bruker APEXII CCD diffractometer
Absorption correction: multi-scan (*SADABS*; Sheldrick, 2008 ▶) *T*_min_ = 0.974, *T*_max_ = 0.989
5966 measured reflections
2895 independent reflections
1963 reflections with *I* > 2σ(*I*)
*R*_int_ = 0.025

#### Refinement

*R*[*F* ^2^ > 2σ(*F* ^2^)] = 0.045
*wR*(*F* ^2^) = 0.116
*S* = 1.03
2895 reflections
196 parameters
7 restraints
H atoms treated by a mixture of independent and constrained refinement
Δρ_max_ = 0.20 e Å^−3^
Δρ_min_ = −0.20 e Å^−3^

### 

Data collection: *APEX2* (Bruker, 2008 ▶); cell refinement: *SAINT* (Bruker, 2008 ▶); data reduction: *SAINT*; program(s) used to solve structure: *SHELXS97* (Sheldrick, 2008 ▶); program(s) used to refine structure: *SHELXL97* (Sheldrick, 2008 ▶); molecular graphics: *SHELXTL* (Sheldrick, 2008 ▶); software used to prepare material for publication: *SHELXTL*.

## Supplementary Material

Crystal structure: contains datablock(s) global, I. DOI: 10.1107/S1600536811021635/ld2011sup1.cif

Structure factors: contains datablock(s) I. DOI: 10.1107/S1600536811021635/ld2011Isup2.hkl

Supplementary material file. DOI: 10.1107/S1600536811021635/ld2011Isup3.cml

Additional supplementary materials:  crystallographic information; 3D view; checkCIF report

## Figures and Tables

**Table 1:** Hydrogen-bond geometry (Å, °)

*D*—H⋯*A*	*D*—H	H⋯*A*	*D*⋯*A*	*D*—H⋯*A*
O5—H5*B*⋯O4^i^	0.85 (1)	1.98 (1)	2.818 (2)	168 (2)
O5—H5*A*⋯O3^ii^	0.85 (1)	1.90 (1)	2.736 (2)	169 (2)
O3—H3⋯O2^ii^	0.84 (2)	1.90 (2)	2.7398 (19)	173 (3)
N1—H1⋯O2^ii^	0.89 (1)	2.20 (1)	2.9865 (18)	146 (2)
N2—H2⋯O1^iii^	0.89 (1)	2.17 (1)	2.9508 (17)	146 (2)
O4—H4⋯O5^iv^	0.85 (2)	1.88 (2)	2.727 (2)	173 (2)

Symmetry codes: (i) 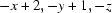; (ii) 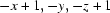; (iii) 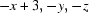; (iv) 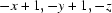.

**Fig. 1.**
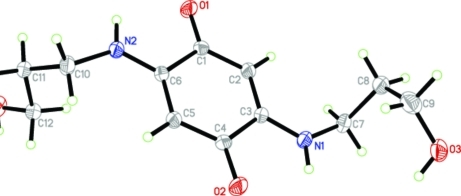
The structure of the title compound. Displacement ellipsoids are drawn at the 30% probability level.

**Fig. 2.**
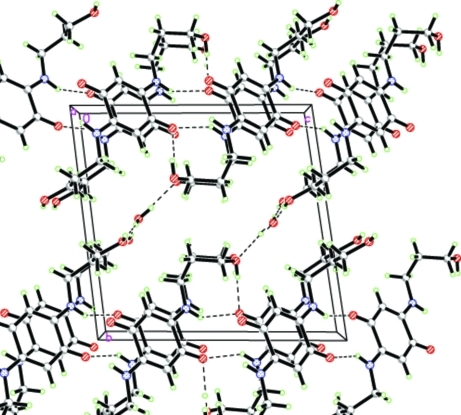
Crystal packing of the title compound.

## supplementary crystallographic information

### Comment

Aminoquinones are used as medicines and herbicides and have interesting redox switching properties. They are formed in the reactions of different amines with quinones or hydroquinone. For example, 1,4-benzoquinone reacts with primary amines to give 2,5-diamino-1,4-benzoquinones. Recently, by reacting hydroquinone with n-propanol amine, 2,5-di[(3-hydroxypropyl)amino]-1,4-benzoquinone has been synthesized. The product was characterized with IR, UV and mass spectrometry, as well as NMR. This and related compounds are of considerable interest since they exhibit potent antitumor and antimalarial activities. However, the single-crystal structure of 2,5-di[(3-hydroxypropyl)amino]-1,4-benzoquinone has not been reported.

### Experimental

Methanol solution (10 ml) of n-propanol amine(2.3 mmol) was added to methanol solution (10 ml) of hydroquinone (0.05 g=0.46 mmol), and was stirred for 0.5 h at room temperature. Then the reaction was refluxed at 50°C for 4 h. A deep-red ropiness crude product was formed. The product was purified by recrystallization from methanol. Long red flat prisms were obtained from methanol solution after vaporizing at room temperature for two weeks.

### Refinement

The structure of the compound was solved with direct methods and then refined anisotropically using full-matrix least-squares procedure. H atoms bonded to N and O atoms were located in a difference Fourier map and refined isotropically with distance restraints O—H = 0.850 and N—H = 0.890 Å. Other H atoms were positioned geometrically and refined using a riding model with C—H = 0.930–0.970 Å and with *U*_iso_(H) = 1.2 (1.5 for methyl groups) times *U*_eq_(C).

### Crystal data

**Table:**

C_12_H_18_N_2_O_4_·H_2_O	*Z* = 2
*M**_r_* = 272.30	*F*(000) = 292
Triclinic, *P*1	*D*_x_ = 1.347 Mg m^−^^3^
Hall symbol: -P 1	Melting point = 437.1–438.3 K
*a* = 4.9272 (8) Å	Mo *K*α radiation, λ = 0.71073 Å
*b* = 11.673 (2) Å	Cell parameters from 1436 reflections
*c* = 11.933 (2) Å	θ = 2.6–26.1°
α = 82.104 (2)°	µ = 0.11 mm^−^^1^
β = 87.994 (2)°	*T* = 296 K
γ = 80.849 (2)°	Strip, red
*V* = 671.13 (19) Å^3^	0.25 × 0.18 × 0.11 mm

### Data collection

**Table:**

Bruker APEXII CCD diffractometer	2895 independent reflections
Radiation source: fine-focus sealed tube	1963 reflections with *I* > 2σ(*I*)
graphite	*R*_int_ = 0.025
phi and ω scans	θ_max_ = 27.0°, θ_min_ = 2.3°
Absorption correction: multi-scan (*SADABS*; Sheldrick, 2008)	*h* = −6→6
*T*_min_ = 0.974, *T*_max_ = 0.989	*k* = −14→14
5966 measured reflections	*l* = −14→15

### Refinement

**Table:**

Refinement on *F*^2^	Primary atom site location: structure-invariant direct methods
Least-squares matrix: full	Secondary atom site location: difference Fourier map
*R*[*F*^2^ > 2σ(*F*^2^)] = 0.045	Hydrogen site location: inferred from neighbouring sites
*wR*(*F*^2^) = 0.116	H atoms treated by a mixture of independent and constrained refinement
*S* = 1.03	*w* = 1/[σ^2^(*F*_o_^2^) + (0.0454*P*)^2^ + 0.0971*P*] where *P* = (*F*_o_^2^ + 2*F*_c_^2^)/3
2895 reflections	(Δ/σ)_max_ < 0.001
196 parameters	Δρ_max_ = 0.20 e Å^−^^3^
7 restraints	Δρ_min_ = −0.20 e Å^−^^3^

### Special details

**Table:**

Geometry. All e.s.d.'s (except the e.s.d. in the dihedral angle between two l.s. planes) are estimated using the full covariance matrix. The cell e.s.d.'s are taken into account individually in the estimation of e.s.d.'s in distances, angles and torsion angles; correlations between e.s.d.'s in cell parameters are only used when they are defined by crystal symmetry. An approximate (isotropic) treatment of cell e.s.d.'s is used for estimating e.s.d.'s involving l.s. planes.
Refinement. Refinement of *F*^2^ against ALL reflections. The weighted *R*-factor *wR* and goodness of fit *S* are based on *F*^2^, conventional *R*-factors *R* are based on *F*, with *F* set to zero for negative *F*^2^. The threshold expression of *F*^2^ > σ(*F*^2^) is used only for calculating *R*-factors(gt) *etc*. and is not relevant to the choice of reflections for refinement. *R*-factors based on *F*^2^ are statistically about twice as large as those based on *F*, and *R*- factors based on ALL data will be even larger.

### Fractional atomic coordinates and isotropic or equivalent isotropic displacement parameters (Å^2^)

**Table:**

	*x*	*y*	*z*	*U*_iso_*/*U*_eq_	
N1	0.6153 (3)	−0.11927 (12)	0.36773 (12)	0.0390 (4)	
N2	1.3092 (3)	0.13162 (11)	0.10709 (11)	0.0351 (3)	
O1	1.2722 (3)	−0.08231 (10)	0.07011 (10)	0.0453 (3)	
O2	0.6703 (3)	0.09049 (10)	0.41065 (10)	0.0552 (4)	
O3	0.5303 (4)	−0.32465 (13)	0.60246 (12)	0.0736 (5)	
O4	1.0113 (3)	0.41209 (12)	−0.14943 (12)	0.0565 (4)	
O5	0.5089 (3)	0.49169 (15)	0.21551 (15)	0.0768 (5)	
C1	1.1245 (3)	−0.04600 (13)	0.14826 (13)	0.0314 (4)	
C2	0.9446 (3)	−0.11051 (13)	0.21472 (13)	0.0340 (4)	
H2A	0.9286	−0.1851	0.1994	0.041*	
C3	0.7922 (3)	−0.06574 (13)	0.30165 (13)	0.0317 (4)	
C4	0.8147 (3)	0.05513 (13)	0.32951 (13)	0.0351 (4)	
C5	0.9895 (3)	0.12030 (13)	0.26347 (13)	0.0349 (4)	
H5	1.0042	0.1949	0.2793	0.042*	
C6	1.1415 (3)	0.07683 (12)	0.17496 (13)	0.0293 (3)	
C7	0.5507 (4)	−0.23474 (14)	0.35927 (14)	0.0396 (4)	
H7A	0.5450	−0.2441	0.2799	0.048*	
H7B	0.3688	−0.2399	0.3915	0.048*	
C8	0.7530 (4)	−0.33485 (14)	0.41825 (14)	0.0435 (4)	
H8A	0.6915	−0.4084	0.4109	0.052*	
H8B	0.9311	−0.3353	0.3809	0.052*	
C9	0.7835 (5)	−0.32661 (18)	0.54126 (16)	0.0589 (6)	
H9A	0.9135	−0.3930	0.5742	0.071*	
H9B	0.8582	−0.2560	0.5486	0.071*	
C10	1.3668 (4)	0.24973 (13)	0.10990 (14)	0.0380 (4)	
H10A	1.5559	0.2458	0.1321	0.046*	
H10B	1.2486	0.2859	0.1662	0.046*	
C11	1.3213 (3)	0.32460 (13)	−0.00383 (13)	0.0339 (4)	
H11A	1.3914	0.3974	−0.0017	0.041*	
H11B	1.4254	0.2842	−0.0612	0.041*	
C12	1.0238 (3)	0.35224 (14)	−0.03697 (14)	0.0382 (4)	
H12A	0.9458	0.2806	−0.0334	0.046*	
H12B	0.9203	0.4011	0.0142	0.046*	
H5A	0.516 (5)	0.4429 (17)	0.2754 (14)	0.092 (8)*	
H5B	0.662 (3)	0.516 (2)	0.205 (2)	0.099 (9)*	
H2	1.405 (4)	0.0890 (14)	0.0584 (13)	0.059 (6)*	
H1	0.535 (4)	−0.0797 (15)	0.4219 (13)	0.063 (6)*	
H4	0.844 (4)	0.437 (2)	−0.167 (2)	0.087 (8)*	
H3	0.456 (5)	−0.2541 (16)	0.601 (2)	0.097 (9)*	

### Atomic displacement parameters (Å^2^)

**Table:**

	*U*^11^	*U*^22^	*U*^33^	*U*^12^	*U*^13^	*U*^23^
N1	0.0467 (9)	0.0316 (7)	0.0382 (8)	−0.0087 (6)	0.0157 (7)	−0.0043 (6)
N2	0.0405 (8)	0.0283 (7)	0.0361 (8)	−0.0068 (6)	0.0112 (6)	−0.0038 (6)
O1	0.0546 (8)	0.0366 (6)	0.0459 (7)	−0.0097 (5)	0.0247 (6)	−0.0129 (5)
O2	0.0752 (9)	0.0391 (7)	0.0519 (8)	−0.0121 (6)	0.0359 (7)	−0.0138 (6)
O3	0.1126 (14)	0.0458 (9)	0.0477 (8)	0.0110 (8)	0.0317 (9)	0.0102 (7)
O4	0.0473 (9)	0.0658 (9)	0.0505 (8)	−0.0093 (7)	−0.0054 (7)	0.0146 (7)
O5	0.0648 (11)	0.0847 (12)	0.0740 (11)	−0.0315 (9)	−0.0232 (9)	0.0418 (9)
C1	0.0345 (9)	0.0279 (8)	0.0299 (8)	−0.0004 (6)	0.0051 (7)	−0.0045 (6)
C2	0.0404 (9)	0.0262 (8)	0.0353 (9)	−0.0063 (7)	0.0074 (7)	−0.0048 (7)
C3	0.0336 (9)	0.0291 (8)	0.0303 (8)	−0.0034 (6)	0.0051 (7)	0.0008 (6)
C4	0.0404 (9)	0.0306 (8)	0.0319 (8)	−0.0007 (7)	0.0103 (7)	−0.0039 (7)
C5	0.0425 (10)	0.0266 (8)	0.0357 (9)	−0.0067 (7)	0.0098 (7)	−0.0057 (7)
C6	0.0295 (8)	0.0271 (8)	0.0290 (8)	−0.0021 (6)	0.0018 (6)	0.0013 (6)
C7	0.0440 (10)	0.0397 (9)	0.0362 (9)	−0.0149 (7)	0.0051 (8)	−0.0001 (7)
C8	0.0511 (11)	0.0349 (9)	0.0438 (10)	−0.0062 (8)	0.0093 (9)	−0.0057 (8)
C9	0.0730 (15)	0.0495 (12)	0.0475 (12)	0.0083 (10)	−0.0057 (11)	−0.0018 (9)
C10	0.0418 (10)	0.0338 (9)	0.0391 (9)	−0.0123 (7)	0.0037 (8)	−0.0014 (7)
C11	0.0351 (9)	0.0292 (8)	0.0371 (9)	−0.0079 (7)	0.0068 (7)	−0.0026 (7)
C12	0.0388 (10)	0.0341 (9)	0.0415 (10)	−0.0073 (7)	0.0050 (8)	−0.0043 (7)

### Geometric parameters (Å, °)

**Table:**

N1—C3	1.3273 (19)	C4—C5	1.392 (2)
N1—C7	1.451 (2)	C5—C6	1.380 (2)
N1—H1	0.891 (9)	C5—H5	0.9300
N2—C6	1.3140 (19)	C7—C8	1.519 (2)
N2—C10	1.456 (2)	C7—H7A	0.9700
N2—H2	0.891 (9)	C7—H7B	0.9700
O1—C1	1.2419 (17)	C8—C9	1.499 (3)
O2—C4	1.2580 (18)	C8—H8A	0.9700
O3—C9	1.422 (2)	C8—H8B	0.9700
O3—H3	0.844 (16)	C9—H9A	0.9700
O4—C12	1.424 (2)	C9—H9B	0.9700
O4—H4	0.850 (16)	C10—C11	1.513 (2)
O5—H5A	0.849 (9)	C10—H10A	0.9700
O5—H5B	0.849 (9)	C10—H10B	0.9700
C1—C2	1.407 (2)	C11—C12	1.504 (2)
C1—C6	1.526 (2)	C11—H11A	0.9700
C2—C3	1.372 (2)	C11—H11B	0.9700
C2—H2A	0.9300	C12—H12A	0.9700
C3—C4	1.515 (2)	C12—H12B	0.9700
			
C3—N1—C7	125.40 (14)	H7A—C7—H7B	107.6
C3—N1—H1	115.3 (13)	C9—C8—C7	112.93 (15)
C7—N1—H1	119.3 (13)	C9—C8—H8A	109.0
C6—N2—C10	126.63 (14)	C7—C8—H8A	109.0
C6—N2—H2	115.3 (12)	C9—C8—H8B	109.0
C10—N2—H2	117.9 (12)	C7—C8—H8B	109.0
C9—O3—H3	108.2 (18)	H8A—C8—H8B	107.8
C12—O4—H4	109.4 (17)	O3—C9—C8	112.68 (18)
H5A—O5—H5B	109.4 (18)	O3—C9—H9A	109.1
O1—C1—C2	124.65 (14)	C8—C9—H9A	109.1
O1—C1—C6	117.67 (13)	O3—C9—H9B	109.1
C2—C1—C6	117.68 (13)	C8—C9—H9B	109.1
C3—C2—C1	121.45 (14)	H9A—C9—H9B	107.8
C3—C2—H2A	119.3	N2—C10—C11	111.84 (13)
C1—C2—H2A	119.3	N2—C10—H10A	109.2
N1—C3—C2	125.71 (15)	C11—C10—H10A	109.2
N1—C3—C4	113.62 (13)	N2—C10—H10B	109.2
C2—C3—C4	120.67 (13)	C11—C10—H10B	109.2
O2—C4—C5	124.28 (15)	H10A—C10—H10B	107.9
O2—C4—C3	117.34 (14)	C12—C11—C10	113.21 (13)
C5—C4—C3	118.38 (13)	C12—C11—H11A	108.9
C6—C5—C4	121.53 (14)	C10—C11—H11A	108.9
C6—C5—H5	119.2	C12—C11—H11B	108.9
C4—C5—H5	119.2	C10—C11—H11B	108.9
N2—C6—C5	126.20 (14)	H11A—C11—H11B	107.7
N2—C6—C1	113.53 (13)	O4—C12—C11	107.74 (13)
C5—C6—C1	120.27 (13)	O4—C12—H12A	110.2
N1—C7—C8	114.31 (14)	C11—C12—H12A	110.2
N1—C7—H7A	108.7	O4—C12—H12B	110.2
C8—C7—H7A	108.7	C11—C12—H12B	110.2
N1—C7—H7B	108.7	H12A—C12—H12B	108.5
C8—C7—H7B	108.7		
			
O1—C1—C2—C3	178.39 (16)	C10—N2—C6—C1	179.18 (14)
C6—C1—C2—C3	−0.9 (2)	C4—C5—C6—N2	179.17 (16)
C7—N1—C3—C2	−0.3 (3)	C4—C5—C6—C1	−0.9 (2)
C7—N1—C3—C4	179.25 (15)	O1—C1—C6—N2	2.2 (2)
C1—C2—C3—N1	179.10 (16)	C2—C1—C6—N2	−178.43 (15)
C1—C2—C3—C4	−0.4 (2)	O1—C1—C6—C5	−177.71 (15)
N1—C3—C4—O2	0.8 (2)	C2—C1—C6—C5	1.7 (2)
C2—C3—C4—O2	−179.63 (16)	C3—N1—C7—C8	83.0 (2)
N1—C3—C4—C5	−178.40 (15)	N1—C7—C8—C9	56.7 (2)
C2—C3—C4—C5	1.2 (2)	C7—C8—C9—O3	58.3 (2)
O2—C4—C5—C6	−179.57 (16)	C6—N2—C10—C11	−126.19 (17)
C3—C4—C5—C6	−0.5 (2)	N2—C10—C11—C12	68.29 (17)
C10—N2—C6—C5	−0.9 (3)	C10—C11—C12—O4	−174.07 (13)

### Hydrogen-bond geometry (Å, °)

**Table:**

*D*—H···*A*	*D*—H	H···*A*	*D*···*A*	*D*—H···*A*
O5—H5B···O4^i^	0.85 (1)	1.98 (1)	2.818 (2)	168 (2)
O5—H5A···O3^ii^	0.85 (1)	1.90 (1)	2.736 (2)	169 (2)
O3—H3···O2^ii^	0.84 (2)	1.90 (2)	2.7398 (19)	173 (3)
N1—H1···O2^ii^	0.89 (1)	2.20 (1)	2.9865 (18)	146.(2)
N2—H2···O1^iii^	0.89 (1)	2.17 (1)	2.9508 (17)	146.(2)
O4—H4···O5^iv^	0.85 (2)	1.88 (2)	2.727 (2)	173 (2)

Symmetry codes: (i) −*x*+2, −*y*+1, −*z*; (ii) −*x*+1, −*y*, −*z*+1; (iii) −*x*+3, −*y*, −*z*; (iv) −*x*+1, −*y*+1, −*z*.
